# Data for identification of porcine X-chromosome inactivation center, XIC, by genomic comparison with human and mouse XIC

**DOI:** 10.1016/j.dib.2015.11.019

**Published:** 2015-11-29

**Authors:** Jae Yeon Hwang, Kwang-Hwan Choi, Chang-Kyu Lee

**Affiliations:** a Department of Agricultural Biotechnology, Animal Biotechnology Major, and Research Institute of Agriculture and Life Science, Seoul National University, Seoul 151-921, Republic of Korea; b Designed Animal & Transplantation Research Institute, Institute of Green Bio Science and Technology, Seoul National University, Gangwon-do 232-916, Republic of Korea

## Abstract

The data included in this article shows homologies of genes in porcine X-chromosome inactivation center, XIC, to each orthologue in human and mouse XIC. Open sequences of XIC-linked genes in human and mouse were compared to porcine genome and sequence homology of each orthologue to porcine genome was calculated. Sequence information of porcine genes encoded in the genomic regions having sequence homology with the human XIC-linked genes and their 2 Kb upstream regions were downloaded. Obtained information was used to design primer pairs for expression and methylation pattern analyses of XIC-linked genes in pigs. The data represented in here is related and applied to the research article entitled *“Dosage compensation of X-chromosome inactivation center, XIC,-linked genes in porcine preimplantation embryos: Non-chromosome wide initiation of X-chromosome inactivation in blastocysts”, published in Mechanisms of Development* Hwang et al., 2015 [1]*.*

## 1 Specifications Table

**Table:** 

Subject area	*Genomics and Genetics*
More specific subject area	Comparative epigenetics/Comparative genomics in mammals
Type of data	Table, Figure, and Text file
How data was acquired	Download public sequence information of human and mouse genes encoded in X-chromosome inactivation center, XIC, to find homologue regions in porcine genome. Sequences of genes encoded in porcine XIC were obtained and used to design primer pairs.
Data format	Analyzed
Experimental factors	None
Experimental features	None
Data source location	Seoul, Republic of Korea
Data accessibility	Data is available in with article.

## 2 Value of the data

• The data is an example for representing severe sequence diversity of non-coding RNA genes in X-chromosome inactivation center, XIC, in eutherians.
• Methods for calculating sequence homology and searching homolog region could provide proper strategies for identifying XIC in eutherians.
• Current data could be used for comparative genomic analyses dealing evolution of XIC in eutherians.
• Designed primer pairs could be efficiently used to analyze expression pattern and methylation pattern of XIC-linked genes and their CpG sites, respectively, in pigs.

## 3 Data

Data shared in the article is sequence homologies of human and mouse X-chromosome inactivation center, XIC, -linked genes to porcine genome and primer pairs for amplifying XIC-linked gene in pigs. And the article contains information such as distribution of CpG sites of four XIC-linked genes and primer pairs examining methylation pattern of the genomic regions in pigs.

## 4 Experimental design, materials and methods

### 4.1 Sequence homology of human and mouse XIC-linked genes to pig genome

Sequence homology of each gene encoded in human and mouse XIC to porcine genome was examined as follows. Genomic and transcript sequences of the human and mouse genes encoded in genomic region ranging between *CDX4* and *RLIM*, which were determined to be the XIC synteny in this study, were downloaded from GenBank and aligned to the pig genome using BLAST. The coverage of individual blast hits (A) was calculated first as follows:

$$\mathrm{Query}\;\mathrm{coverage}(A)=\frac{\mathrm{Length}\;\mathrm{of}\;\mathrm{each}\;\mathrm{blast}\;\mathrm{hit}\;\mathrm{in}\;\mathrm{query}}{\mathrm{Query}\;\mathrm{Length}}$$

The sequence homology rate of each blast hit (B) from alignment of one query was calculated by magnifying the sequence identity between blast hits and their searched counterpart region in pig:

Homologyrateofindividualblasthit(B)=Qureycoverageofblasthit(A)×Identity

After calculating the homology rate of each blast hit (*B*) from the alignment of one query, their sum was considered to be the homology rate of the query (*C*), which is a partial fragment of the genomic sequence or transcript sequence of human and mouse XIC-linked genes.

Homologyrateofquery(C)=SumofHomologyrateofindividualblasthit(B)

The homology rate of XIC-linked genes (*D*) of which partial fragment sequences were used for query, was calculated by averaging the homology rate of each query (*C*):

Homology rate of gene(D)=Average of Homology rate of query(C)

**Fig. 1:**
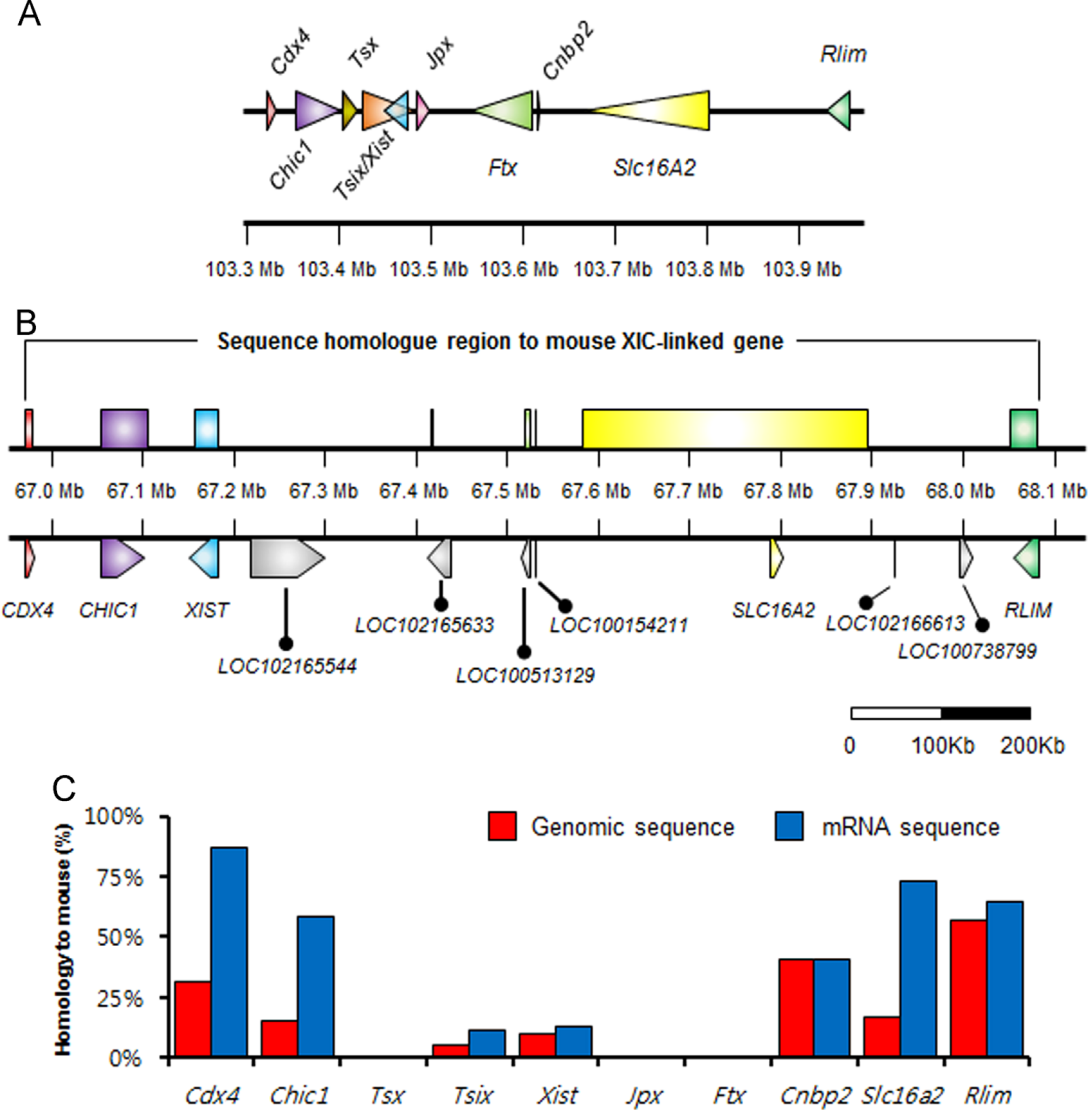
Mouse XIC and sequence comparison between porcine and mouse XIC-linked genes**.** (A) Mouse XIC diagram. Arrows indicate mouse genes located in the XIC. The diagram is scaled and mouse genomic location was written at bottom with 100 kb intervals. (B) Candidate porcine XIC and the genomic region homologs to mouse XIC-linked genes. The porcine genomic regions having homology with mouse genes are represented with boxes that are the same color as their counterpart mouse genes. And currently annotated genes in the genomic region were represented on the bottom with arrows. The diagram is scaled and porcine genomic location was shown as same to Fig. 1B. (C) Sequence homology rate between mouse XIC-linked genes and their counterparts in the pig genome. Blue and red boxes represent the homologies of transcript and genomic sequences of mouse XIC-linked genes, respectively, to counterpart sequences in porcine genome.

**Fig. 2:**
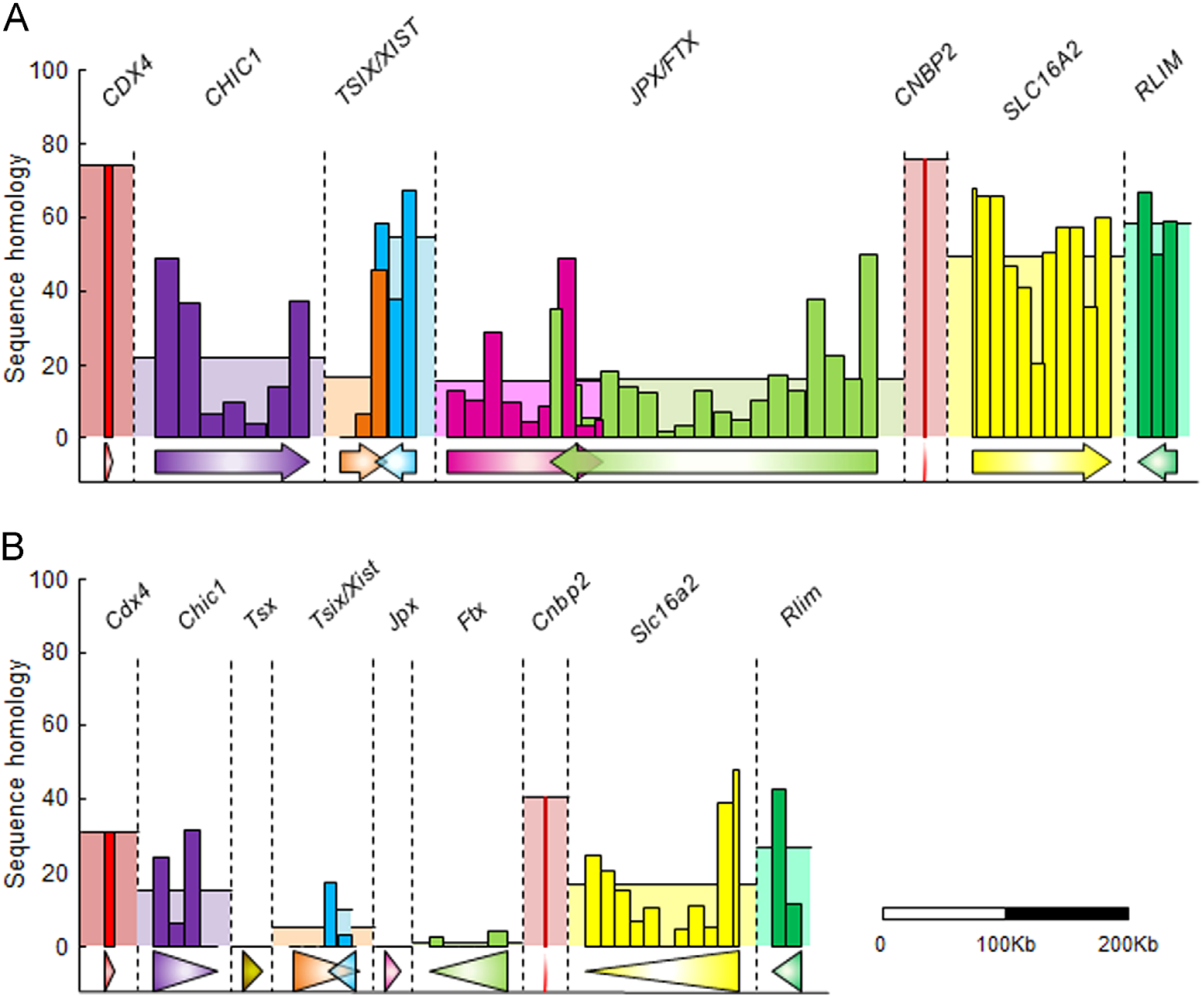
Sequence homology of the human and mouse XIC-linked gene locus with counterparts on the porcine genome. Genomic sequences of human (A) and mouse (B) XIC-linked genes were compared to their counterparts in the pig genome. Genes are represented by arrows and genes of the same color mean they are orthologous between human and mice. Bars and rectangles on the arrows represent the sequence homology of each fragment sequences of the gene and their average homology, respectively (see Supplementary materials and methods). Arrow width indicates the genomic length of the gene.

Sequence homologies of human and mouse XIC-linked genes to porcine genome were calculated according to equations (Fig. 1, Fig. 2, and Supplementary materials 1–4).

### 4.2 Primer pairs detecting porcine XIC-linked genes

Primer pairs for detecting and analyzing expression of the porcine XIC-linked genes were designed by using Primer 3 plus web-service [2]. Primer pairs for RT-PCR (Table 1) and Realtime PCR (Table 2) was prepared one another. The lengths of RT-PCR products ranged approximately from 450 bp to 700 bp, except *ACTB* (131 bp), which were used for reference genes, and uncharacterized gene, *LOC102166613* (115 bp). Primer pairs for Realtime PCR were designed to amplify PCR product ranging in length between 90 bp and 183 bp. Designed primer pairs were validated by sequencing amplicons and used for expression analyses in the related study [1]. The primer pairs will be used for the expression analyses of porcine XIC-linked genes.

### 4.3 Distribution of CpG sites of XIC-linked genes and primer pairs for bisulfite sequencing

**Fig. 3:**
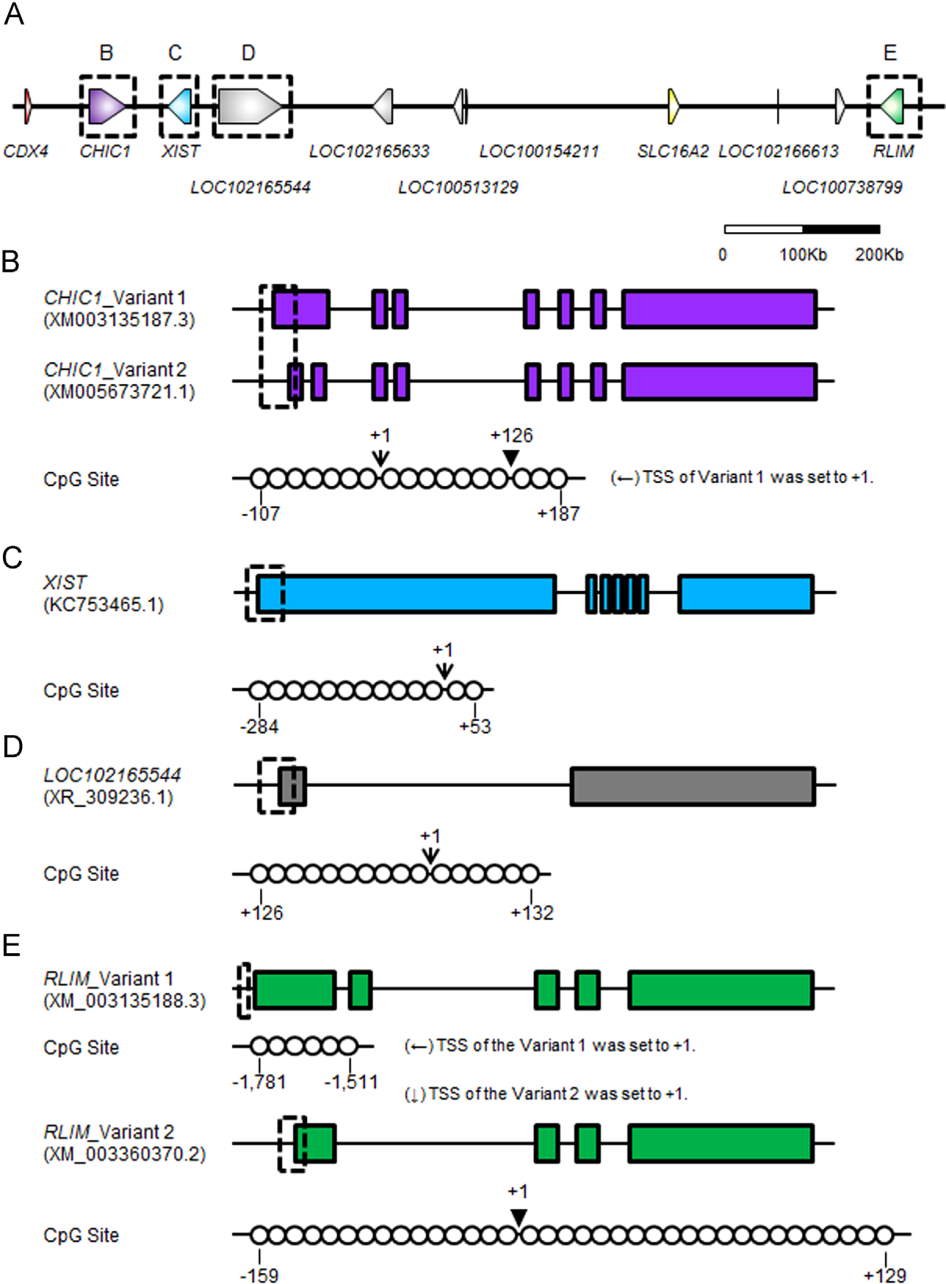
Diagram of CpG sites of genes in the porcine XIC. (A) Diagram of the porcine XIC. The diagram is scaled. (B–E) Gene structure and identified CpG sites of each gene. The structure of *CHIC1* (B), *XIST* (C), *LOC10216554* (D), and *RLIM* (E) is depicted. A rectangle indicates the exon of the gene and circles represent CG dinucleotides. Numbers under the circles or on the arrows and arrowheads indicates genomic distance of them from the transcription starting sites of each gene. Arrows and arrowheads indicate the first and second transcription starting sites, respectively. The CpG sites of the *RLIM* variant 1 was considered not to be a proper differentially methylated region because the region was identically methylated in both sexes of PEFs (data not shown).

Distribution of CG dinucleotide were searched manually around the transcription starting sites, TSSs, of four XIC-linked genes, *CHIC1*, *XIST*, uncharacterized gene *LOC102165544* and *RLIM* (Fig. 3) to design primer pairs for examining promoter methylation patterns of the genes. Primer pairs which could amplify the genomic region containing CG dinucleotide were prepared (Table 3). The length of PCR product amplified by primer pairs targeting CpG sites of *CHIC1*, *XIST*, *LOC102165544* and *RLIM* were 360 bp, 429 bp, 290 bp and 348 bp, respectively. And also, the numbers of CG dinucleotide in the four amplicon were 17, 13, 16, and 31, respectively. Primer pairs amplifying CpG sites of XIST gene was one of the primer set designed previously [3] and primers targeting genomic sites of remaining three genes were designed newly in here. Efficient working of the primer pairs was validated in the related study [1].

## Appendix A Supplementary material

Supplementary material **Supplementary material 1. Genomic sequence homology of human XIC-linked genes to porcine genome.**

**Supplementary material 2. Transcript sequence homology of human XIC-linked genes to porcine genome.**

**Supplementary material 3. Genomic sequence homology of mouse XIC-linked genes to porcine genome.**

**Supplementary material 4. Transcript sequence homology of mouse XIC-linked genes to porcine genome.**

## Footnotes

Appendix A Supplementary data associated with this article can be found in the online version at doi:10.1016/j.dib.2015.11.019.
