# Supplementary material for: Data for identification of porcine X-chromosome inactivation center, XIC, by genomic comparison with human and mouse XIC
Source: Data Brief. 2015 Nov 29;5:1072–7. doi: 10.1016/j.dib.2015.11.019 (PMC4689114; doi:10.1016/j.dib.2015.11.019)
Supplement: Supplementary file 1 [file mmc1.zip › Supplement/Hwang_et_al_2015_DIB_Table_1.docx]

| Table 1. Primer list used for RT-PCR | | | |
| --- | --- | --- | --- |
| Gene symbol | Primer sequence (5´ to 3´) | | Amplicon size |
| *CDX4* | F | CCTCGGGAAGACTGGAAC | 602 bp |
|  | R | TGCTGTATCTCAATAGGCTGAAA |  |
| *CHIC1* | F | TGTCTCAACAAAAGAACCAGAAGA | 645 bp |
|  | R | AAAAGCAGGTAAGAGTAAAACCATT |  |
| *XIST* | F | GCAGCTCTAAGAAGTTCCGCATT | 474 bp |
|  | R | TGTCCAGTTATCCCAAGGCATCT |  |
| *LOC102165544* | F | CTAAGATGGCGGCGTTTG | 666 bp |
|  | R | TGGGTTTATTTCTGGGCTTT |  |
| *LOC102165633* | F | TCAAGTTTTCACCACAAATACCA | 788 bp |
|  | R | GGCATACAGGGACCAGAGAA |  |
| *LOC100513129* | F | CACCAAGCTCGATCAGAAAC | 688 bp |
|  | R | GAGGAAGCACTAGGTGGAAGAA |  |
| *LOC100154211* | F | TGCCCCATTGAAACTACCAC | 500 bp |
|  | R | CCCTCCCCCATACACACTC |  |
| *SLC16A2* | F | ACCCCAAGCAAGAGAGGTGT | 646 bp |
|  | R | TCAGAGGGACGAACAAGAGG |  |
| *LOC102166613* | F | TCGGTTGGATGGTGGTCT | 115 bp |
|  | R | CGATTTTTCTCTCTTTCCTCCT |  |
| *RLIM* | F | CCCACCACCGCAAAACTC | 567 bp |
|  | R | CTTCCCTCAGTCTCATTTACCA |  |
| *ACTB* | F | GTGGACATCAGGAAGGACCTCTA | 131 bp |
|  | R | ATGATCTTGATCTTCATGGTGCT |  |
