# Supplementary material for: Data for identification of porcine X-chromosome inactivation center, XIC, by genomic comparison with human and mouse XIC
Source: Data Brief. 2015 Nov 29;5:1072–7. doi: 10.1016/j.dib.2015.11.019 (PMC4689114; doi:10.1016/j.dib.2015.11.019)
Supplement: Supplementary file 1 [file mmc1.zip › Supplement/Hwang_et_al_2015_DIB_Table_2.docx]

| Table 2. Primer pairs used in Realtime PCR | | | |
| --- | --- | --- | --- |
| Gene symbol | Primer sequence (5’ to 3’) | | Amplicon size |
| *CDX4* | F | AGCCCCTATGCGTGGATG | 154 bp |
|  | R | GCTCTGATTTTCTCCGAATG |  |
| *CHIC1* | F | AATGTGAAATGGCTGCTGTG | 97 bp |
|  | R | ATCTTCTGGTTCTTTTGTTGAGA |  |
| *XIST* | F | GCTCCAACCAATCTAAAAGGA | 131 bp |
|  | R | ATGCCCCATCTCCACCTAA |  |
| *LOC102165544* | F | CTAAGATGGCGGCGTTTG | 135 bp |
|  | R | TTGTTTTTCAGGGAATAGAGAGG |  |
| *LOC102165633* | F | GTCTGGGGTTTGTTCCTGTG | 149 bp |
|  | R | GGCTGTAGTCATCCTCTGATTTTT |  |
| *LOC100513129* | F | GCACCCTTCACCCAGTCTT | 129 bp |
|  | R | CGCAGGGCTCAATATACCTC |  |
| *LOC100154211* | F | CCACCTCCTTTTGCTGATTG | 133 bp |
|  | R | TTCCCAGTCCTTTCCTCTTTT |  |
| *SLC16A2* | F | TGGTGAGGAAGACAAGGATG | 183 bp |
|  | R | CCAGGAGCAGGAAGGAGATG |  |
| *LOC102166613* | F | TCGGTTGGATGGTGGTCT | 115 bp |
|  | R | CGATTTTTCTCTCTTTCCTCCT |  |
| *RLIM* | F | CCCACCACCGCAAAACTC | 159 bp |
|  | R | CGGCTCACTGCTCTCCAA |  |
| *G6PD* | F | TTCTTTGCCCGCAACTCCTA | 90 bp |
|  | R | GCGTTCATGTGGCTGTTGAG |  |
| *HPRT1* | F | CATTATGCCGAGGATTTGGAA | 90 bp |
|  | R | CTCTTTCATCACATCTCGAGCAA |  |
| *H19* | F | CTCAAACGACAAGAGATGGT | 122 bp |
|  | R | AGTGTAGTGGCTCCAGAATG |  |
| *RN18S* | F | ACAAATCGCTCCACCAACTAAGA | 90 bp |
|  | R | CGGACACGGACAGGATTGAC |  |
| *ACTB* | F | GTGGACATCAGGAAGGACCTCTA | 131 bp |
|  | R | ATGATCTTGATCTTCATGGTGCT |  |
| *YWHAG* | F | CAGCCCGTGAAGATGGTG | 130 bp |
|  | R | CATTGGACAGTGGCTCATTC |  |
