# Supplementary material for: Data for identification of porcine X-chromosome inactivation center, XIC, by genomic comparison with human and mouse XIC
Source: Data Brief. 2015 Nov 29;5:1072–7. doi: 10.1016/j.dib.2015.11.019 (PMC4689114; doi:10.1016/j.dib.2015.11.019)
Supplement: Supplementary file 1 [file mmc1.zip › Supplement/Hwang_et_al_2015_DIB_Table_3.docx]

| Supplementary table 3. Primer pairs used in bisulfite-treated gDNA amplification | | | | |
| --- | --- | --- | --- | --- |
| Gene | Primer | Primer sequence (5´ to 3´) | Temp | Amplicon size |
| *CHIC1* | Outer-F | TTTTTGTTTTGGAGGGAAG | 54°C | 400bp |
|  | Outer-R | TACTCATCACATAACCCCTTAAT |  |  |
|  | Inner-F | AGGGAAGGGGGTTATAATTT | 54°C | 360bp |
|  | Inner-R | CCACCCTCACAAAAACTCTA |  |  |
|  |  |  |  |  |
| *XIST* | Outer_F | TGGTTAAATGAGGTATTTGGA | 54°C | 525bp |
|  | Outer_R | CCATAAAACATAACTAAAAACTAAA |  |  |
|  | Inner_F | TTTGTTATATTGTTTGTGGAAAA | 50°C | 429bp |
|  | Inner_R | CCATAAAACATAACTAAAAACTAAA |  |  |
|  |  |  |  |  |
| *LOC102165544* | Outer-F | TAAAAGAAATTTGGGATGGA | 56°C | 342bp |
|  | Outer-R | ATTCCCAAACCTTCCTTAAC |  |  |
|  | Inner-F | AATTTTAGGGGTGAGAAAGG | 56°C | 290 bp |
|  | Inner-R | ATTCCCAAACCTTCCTTAAC |  |  |
|  |  |  |  |  |
| *RLIM* | Outer-F | TTTTTGATTATTAGTGAGGTTGAA | 52°C | 503 bp |
|  | Outer-R | TTTCTTCCCCTAAAACCCCTTT |  |  |
|  | Inner_F | TTTGTTGTGGTTTAGTAGTAATAATTT | 52°C | 348 bp |
|  | Inner_R | AAACCCCTTTATAAATCAAAA |  |  |
