# Supplementary figures and images for: Data for identification of porcine X-chromosome inactivation center, XIC, by genomic comparison with human and mouse XIC
Source: Data Brief. 2015 Nov 29;5:1072–7. doi: 10.1016/j.dib.2015.11.019 (PMC4689114; doi:10.1016/j.dib.2015.11.019)

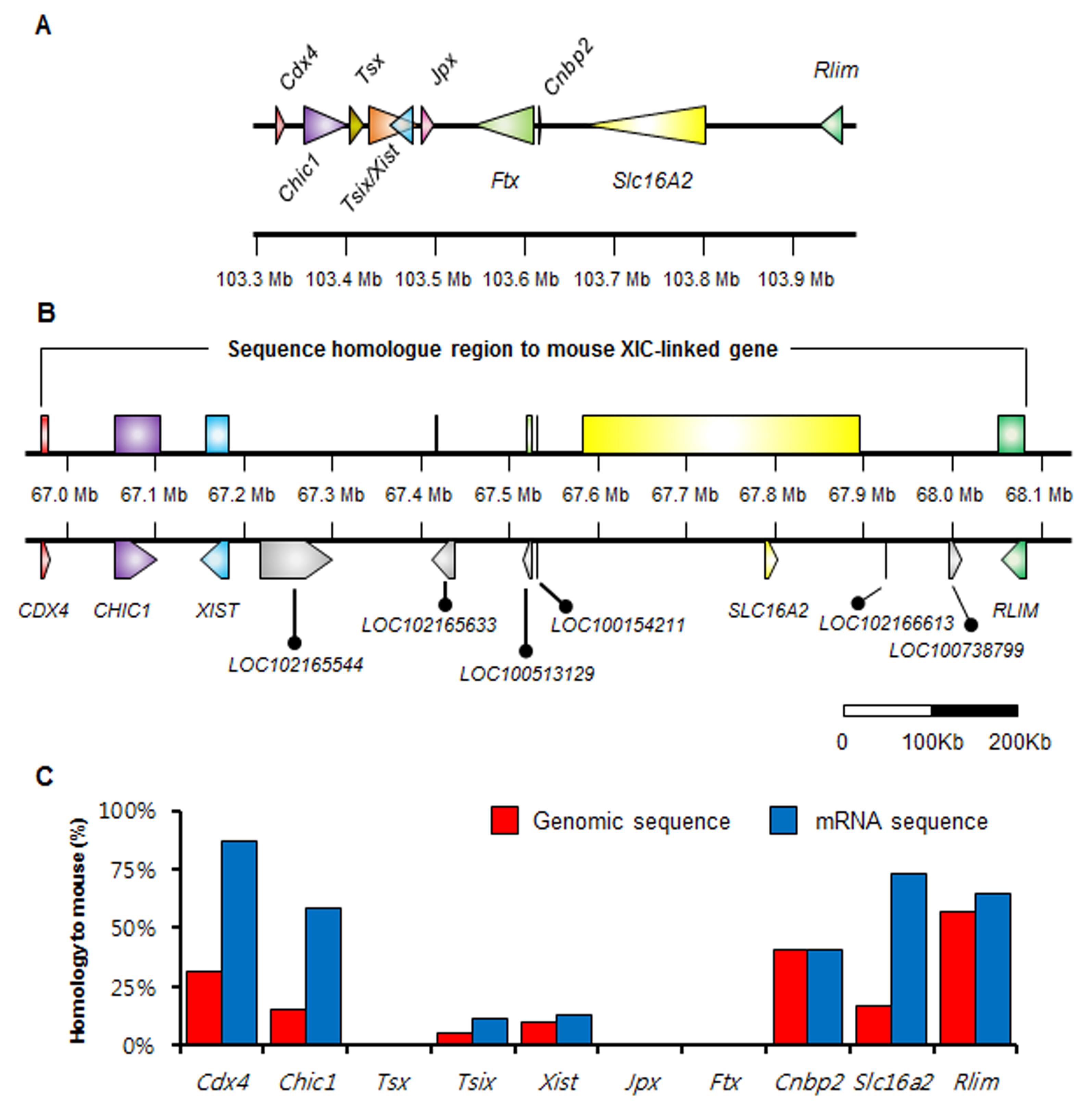

Supplement: Supplementary file 1 [file mmc1.zip › Supplement/Hwang_et_al_2015_DIB_Fig1.jpg]

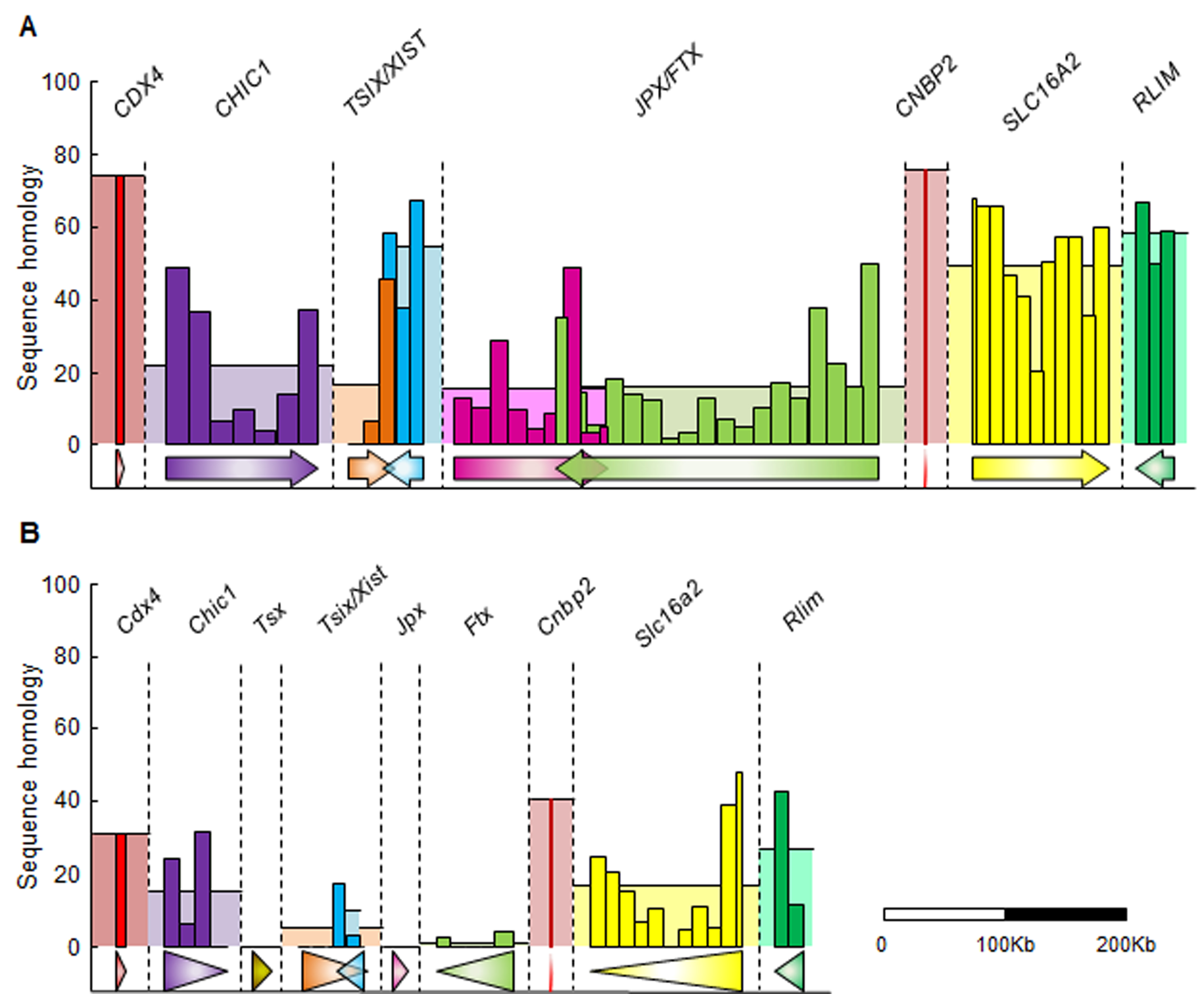

Supplement: Supplementary file 1 [file mmc1.zip › Supplement/Hwang_et_al_2015_DIB_Fig2.jpg]

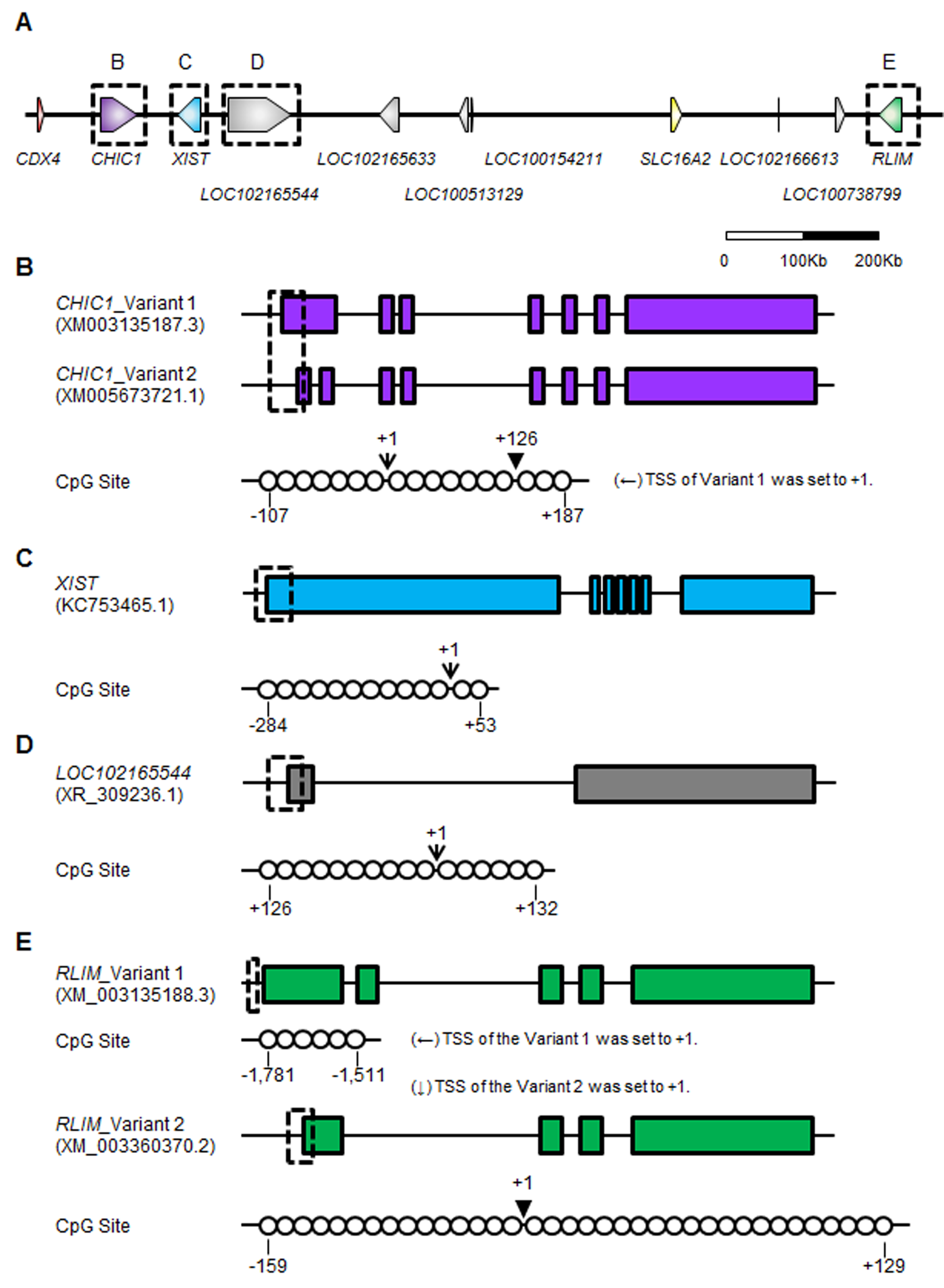

Supplement: Supplementary file 1 [file mmc1.zip › Supplement/Hwang_et_al_2015_DIB_Fig3.jpg]
